# IgG4-Related disease with diffuse myopericardial involvement- value of CMR: a case report and literature review of cardiac involvement

**DOI:** 10.1186/s12872-024-03874-3

**Published:** 2024-04-06

**Authors:** Golnaz Houshmand, Najme-Sadat Moosavi, Amirhossein Shahbazkhani, Hamidreza Pouraliakbar

**Affiliations:** 1 Rajaie Cardiovascular Medical and Research Centre, Iran University of Medical Sciences, Tehran, Iran; 2 Tehran Heart Center, Tehran University of Medical Sciences, Tehran, Iran; 3 Department of Radiology, Shariati Hospital, Tehran University of Medical Sciences, Tehran, Iran

**Keywords:** IgG4-related disease, Cardiovascular system, Restrictive cardiomyopathy, Constrictive pericarditis

## Abstract

**Background:**

IgG4-related disease is a fibro-inflammatory disorder with an unknown etiology, which can affect multiple organ systems, including the cardiovascular system. While most reported cases of cardiovascular involvement are primarily associated with the aorta, there have been sporadic reports of isolated cardiac involvement.

**Case presentation:**

This paper presents a documented case of IgG4-related systemic disease with symptoms indicative of restrictive cardiomyopathy. Subsequent Cardiac Magnetic Resonance imaging revealed diffuse myopericardial involvement, characterized by pericardial thickening and enhancement, accompanied by subepicardial and myocardial infiltration. Considering the rarity of cardiac involvement in our case, we conducted a thorough review of the existing literature pertaining to various patterns of cardiac involvement in IgG4-related disease, as well as the diagnostic modalities that can be employed for accurate identification and assessment.

**Conclusions:**

This case report sheds light on the importance of recognizing and evaluating cardiac manifestations in IgG4-related systemic disease to facilitate timely diagnosis and appropriate management.

## Background

Immunoglobulin G4-related disease (IgG4-RD) is a distinctive fibro-inflammatory disorder driven by immune-mediated processes, characterized by the infiltration of IgG4-positive plasma cells and elevated levels of serum IgG4. This condition often leads to diffuse fibrosis and tumefactive lesions within affected organs. The pancreas, bile ducts, salivary and lacrimal glands, retroperitoneum, and kidney are commonly involved in this disorder [1]. While vascular manifestations, including aortitis, arteritis, periaortitis, periarteritis, and inflammatory aneurysms, have been well-documented [2], isolated cardiac involvement remains less frequently reported in the medical literature.

Accurate diagnosis of IgG4-RD necessitates a comprehensive evaluation, considering clinical, serological, radiological, and pathological features while ruling out other disorders that may mimic its presentation [3]. Owing to the non-specific and variable nature of its manifestations, patients often undergo assessments by multiple healthcare professionals, leading to potential delays in diagnosis [4].

Given the potential for organ damage, timely identification of IgG4-related disease assumes critical significance, as it enables prompt initiation of appropriate treatment measures, ultimately leading to improved patient outcomes.

## Case presentation

In 2014, a 60-year-old male patient presented with a noticeable bulge in his neck, which resembled an enlarged lymph node. An excisional biopsy was performed, but subsequent pathology tests yielded reactive lymph node, failing to ascertain the cause of the bulge. Over the following years, the patient experienced a range of symptoms, including anemia, abdominal bloating, and a gradual weight loss of approximately 6 kg. In 2017, he reported nocturnal headache and neck swelling. Further investigations were conducted on that time, including a comprehensive computed tomography (CT) scan covering the neck, chest, abdomen, and pelvis. The CT scan revealed diffuse lymphadenopathy in multiple areas, as well as the presence of soft tissue density masses around the abdominal aorta, pericardial effusion, and left-sided pleural effusion. Echocardiography showed mild pericardial effusion and mild pericardial thickening. A pleural fluid tap was performed, and tests for tuberculosis and non-tuberculosis infections returned negative results. Furthermore, cervical lymph nodes were excised, and pathological examination revealed reactive lymph nodes. As the results remained inconclusive, a positron emission tomography (PET) scan was conducted to obtain more detailed information. The PET scan revealed numerous faintly/non-2-fluoro-2-deoxy-D-glucose (FDG) avid lymph nodes in the cervical, thoracic, retroperitoneal, and pelvic regions, along with FDG avid infiltrations observed in the gastrohepatic and left para-aortic regions. Following this, a biopsy of the retroperitoneal mass was performed. Immunohistochemical study of the biopsy sample indicated dense lymphoplasmacytic infiltrate with IgG4 positive plasma cells and storiform fibrosis. Subsequent measurement of serum IgG4 level showed a more than fivefold increase than the normal range (730 mg/dl).

The biopsy and serologic results established a definitive diagnosis of IgG4-RD. The patient's treatment approach was adjusted accordingly, and he underwent a four-month course of prednisolone(40 mg/day) and two doses of 1000 mg at 2-weekly intervals, and four doses of 375 mg/m2 at weekly intervals of rituximab, continued with a maintenance dose of prednisolone(5 mg/daily) combined with azathioprine (75 mg/daily) and methotrexate (2.5 mg,6 tablets per week). This therapeutic regimen significantly improved his symptoms, reflecting a positive response to the treatment.

**Fig. 1:**
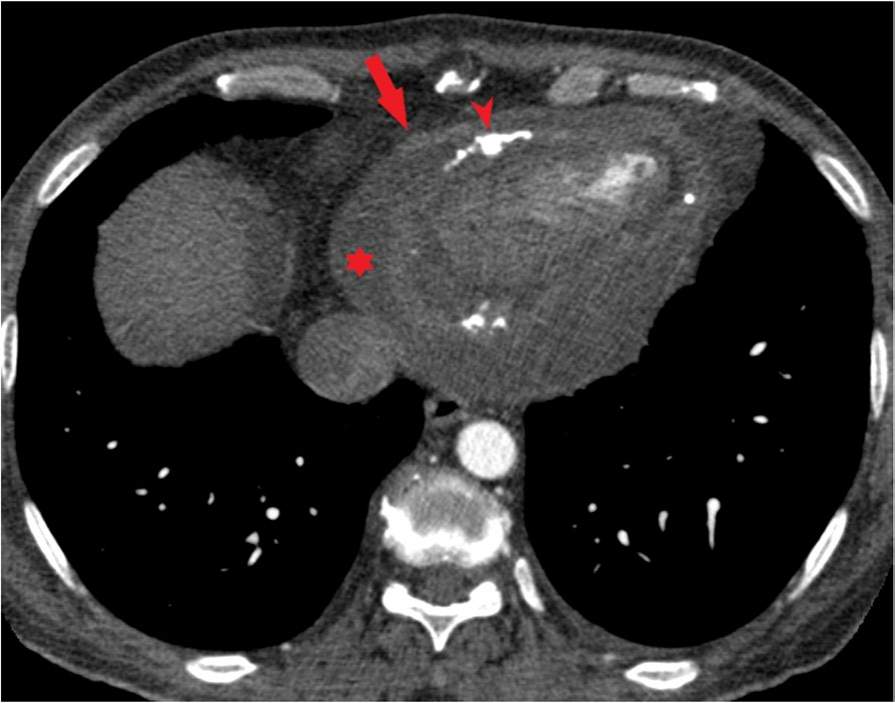
Contrast enhanced chest CT scan shows pericardial effusion (asterisk) with diffuse enhancing pericardial thickening (arrow). Areas of pericardial calcification are visible (arrowhead)

**Fig. 2:**
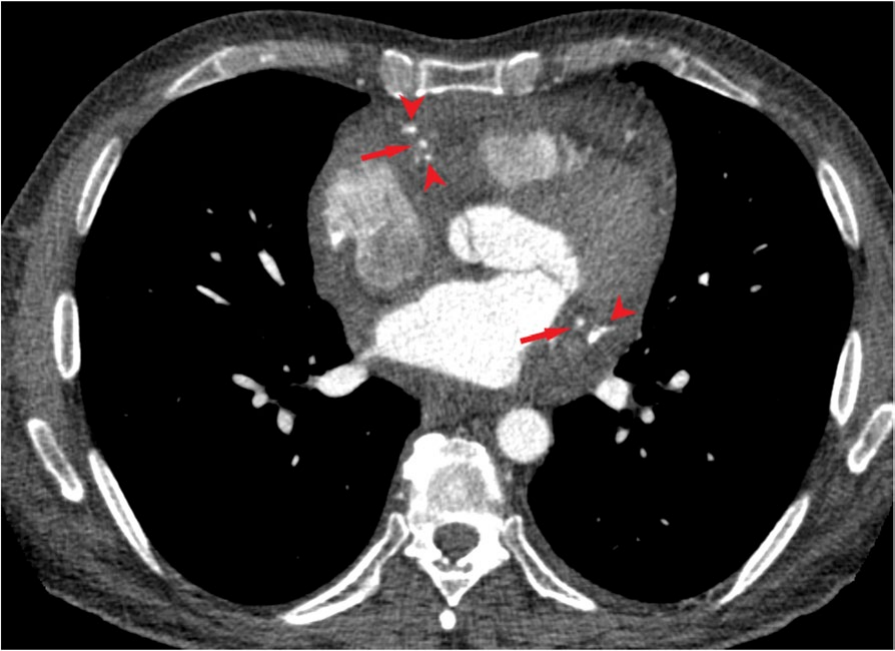
Contrast enhanced chest CT scan shows periarterial soft tissue infiltration around the coronary arteries (arrow) with foci of calcification (arrowhead)

**Fig. 3:**
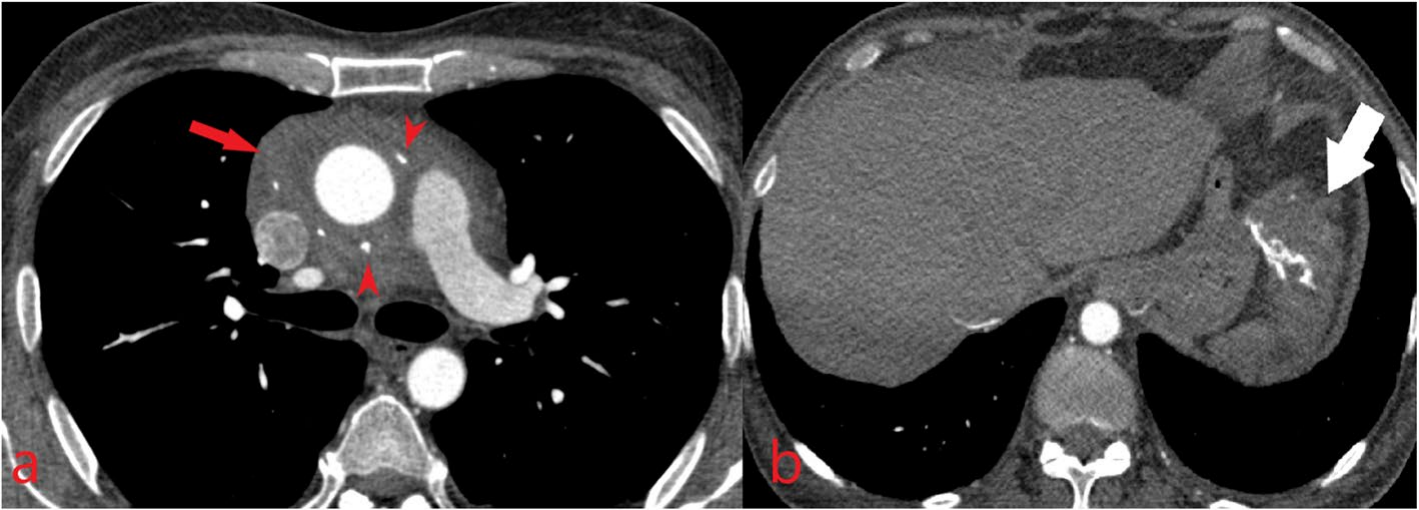
Contrast enhanced chest CT scan shows **3a**: Mediastinal soft tissue infiltration (red arrow) with foci of calcification (red arrowhead) and **3b**: Calcified soft tissue mass in upper abdomen (white arrow)

In follow-up contrast enhanced chest CT scan in 2020, mild pericardial effusion with diffuse pericardial thickening was seen containing scattered foci of calcification. (Fig. 1) Also there was periarterial soft tissue infiltration with areas of calcification around the coronary arteries. (Fig. 2) Diffuse mediastinal infiltration with foci of calcification and calcified soft tissue mass in upper abdomen were also observed. (Fig. 3) As it is evident, the appearance and pattern of involvement in the cardiac and mediastinum were similar to the biopsied retroperitoneal lesion.

Despite the initial positive response to treatment, in 2023, the patient experienced dyspnea and leg edema. The serum IgG4 level was 295 mg/dl which had decreased compared to the time of diagnosis, but was still higher than the normal level. Echocardiography revealed pericardial effusion with left ventricular (LV) dysfunction, biatrial enlargement, and septal bounce, and the patient was referred for Cardiac Magnetic Resonance imaging (CMR) for further evaluation.

**Fig. 4:**
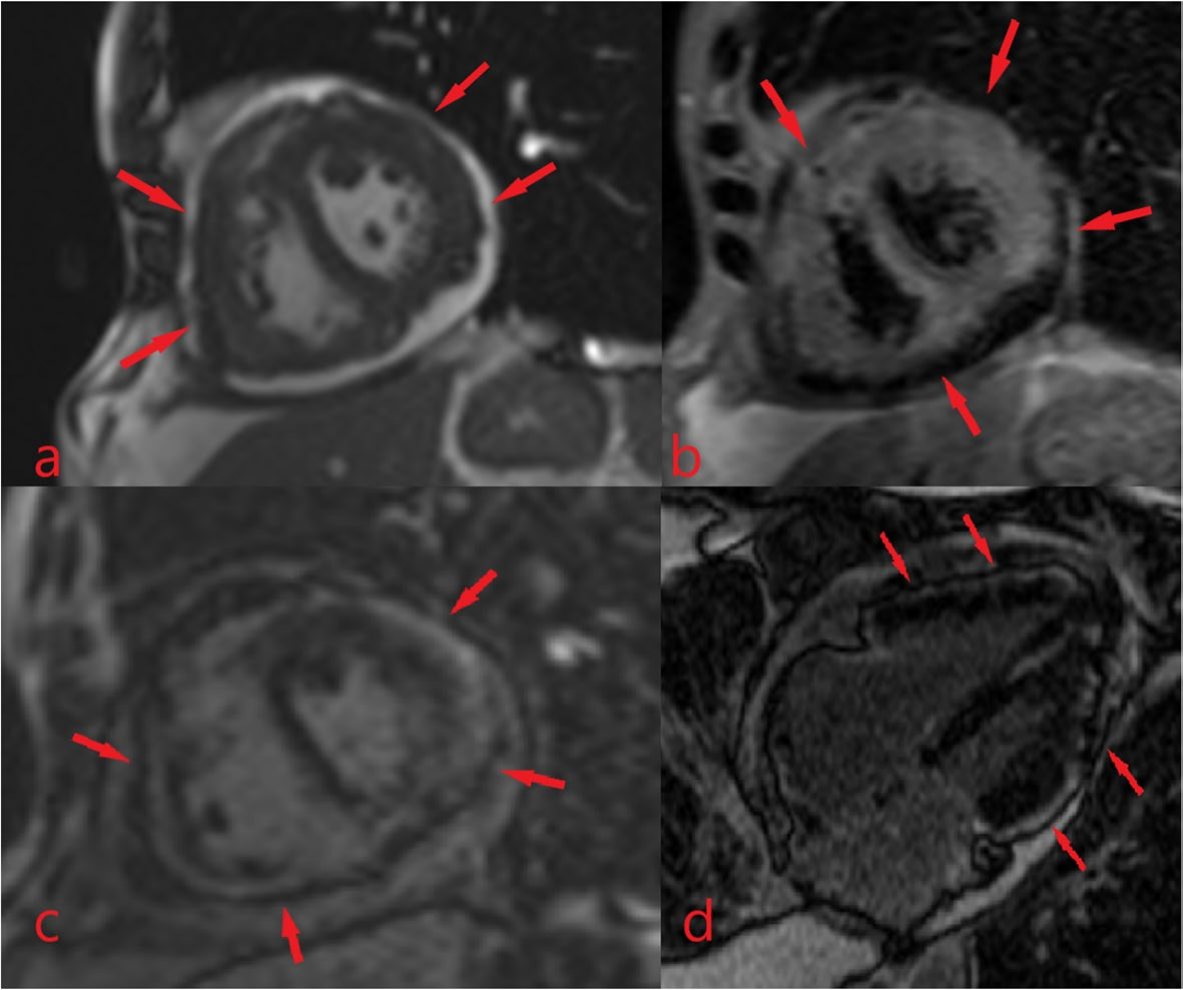
Cardiac magnetic resonance imaging shows **4a**: Diffuse subepicardial infiltrative lesion in Cine image with **4b:** increased T2 weighted signal intensity in Short tau inversion recovery image.**4c and 4d**: in late gadolinium enhancement images there is a diffuse subepicardial enhancement in both LV and RV myocardium

**Fig. 5:**
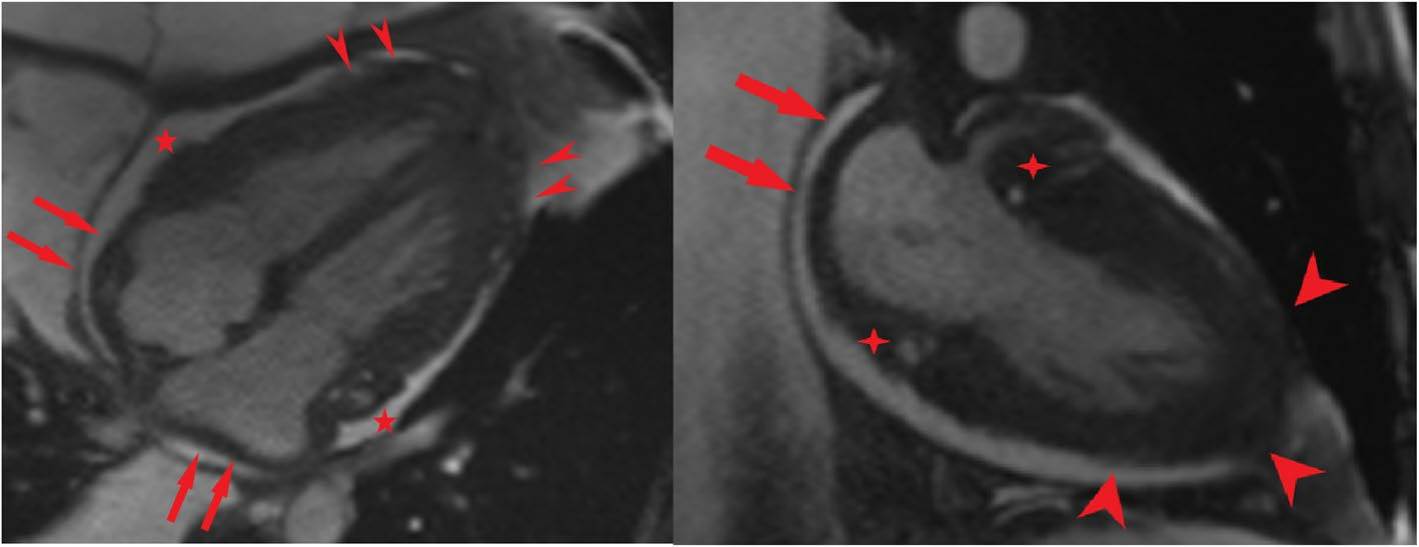
Cardiac magnetic resonance imaging shows moderate pericardial effusion and mild diffuse thickening of the parietal pericardium and infiltrative irregular enhancing thickening of the visceral pericardium (arrowhead). There are diffuse infiltrative thickening of the bi-atrial walls (arrow) and periarterial infiltration around the coronary arteries (asterisk)

The CMR revealed small bi-ventericular size (LVEDVI = 36 ml/m2, RVEDVI = 48 ml/m2) and ejection fraction (LV EF = 45%, RV EF = 37%). In Cine and Short tau inversion recovery images, there was a diffuse subepicardial infiltrative lesion with increased T2 weighted signal intensity. In late gadolinium enhancement images(LGE), there was a diffuse subepicardial enhancement in both LV and RV myocardium, suggesting non-ischemic myocardial injury (Fig. 4). There was mild diffuse thickening of the parietal pericardium and infiltrative irregular enhancing thickening of the visceral pericardium. Moderate circumferential pericardial effusion was also evident. Evidence of constrictive physiology was observed as septal bouncing and respiratory phasic interventricular interdependency. Furthermore, diffuse infiltrative thickening of the bi-atrial walls and periarterial infiltration around the coronary arteries were also observed. (Fig. 5) No valvular abnormality was found.

These findings point to diffuse involvement of the pericardium and myocardium, suggesting a combination of constrictive pericarditis and restrictive cardiomyopathy, all occurring in the context of IgG4-RD.

**Fig. 6:**
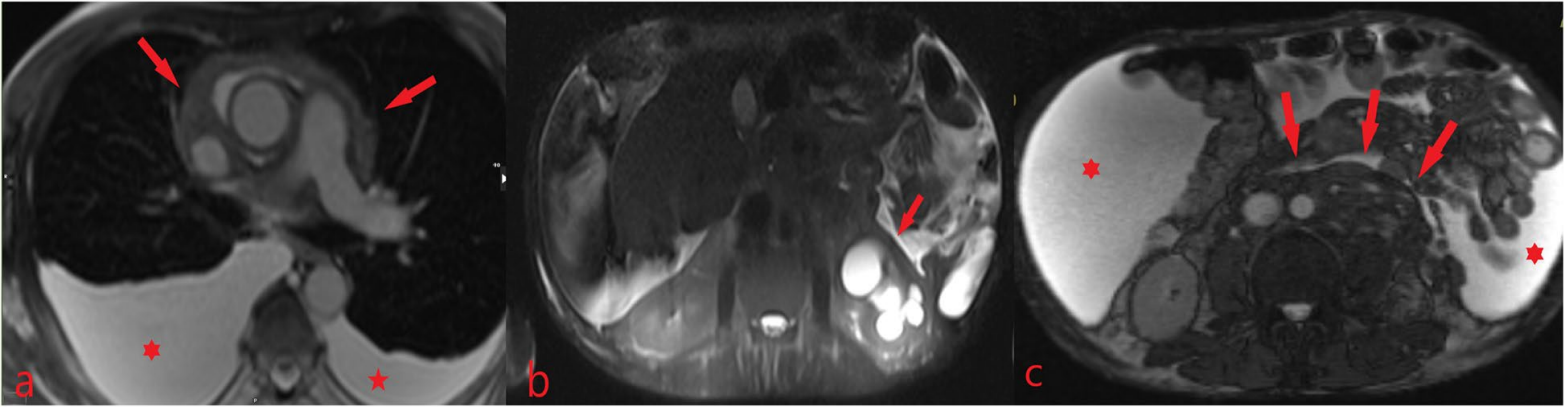
Magnetic resonance imaging shows** 6a**: bilateral pleural effusion and mediastinal infiltration involving vascular structures,**6b**: hydronephrosis and atrophic changes in the left kidney and **6c**: ascites and retroperitoneal infiltration

The CMR also revealed mediastinal infiltration involving vascular structures, indicating the spread of the disease to the surrounding tissues. Furthermore, the CMR demonstrated significant extracardiac manifestations of IgG4-RD, including bilateral pleural effusion, mass-like lesions in both cardiophrenic angles, ascites, and retroperitoneal infiltration resulting in hydronephrosis and atrophic changes in the left kidney. (Fig. 6).

These findings underscore the multisystem involvement and relapsing nature of IgG4-RD, even after initial treatment. The CMR results are valuable for guiding further management and treatment decisions to address the complex and widespread effects of the disease.

## Discussion

IgG4-RD is a group of conditions characterized by common clinical, serologic, and histopathologic features. It was first recognized in 2001 when patients with autoimmune pancreatitis were found to have elevated IgG4 serum concentrations [5]. Subsequently, in 2003, Kamisawa et al. introduced the concept of IgG4-related autoimmune disease [6]. Since then, extensive research has been conducted to explore the various presentations of this condition.

Three major criteria were proposed for diagnosing IgG4-RD [7]: (1) Diffuse or localized swelling or masses in one or more organs on clinical examination. (2) Elevated serum IgG4 concentrations (≥ 135 mg/dL). (3) Marked lymphoplasmacytic infiltration, storiform fibrosis, and organ infiltration by IgG4-positive plasma cells at the histopathologic examination.

The diagnosis is considered definite when all three criteria are met, probable when the first and third criteria are present, and possible when the first and second criteria coincide.

IgG4-RD can affect nearly every organ system, and recognition of its involvement in the cardiovascular system has led to the term "IgG4-related cardiovascular disease (CVD)" used by some researchers [7].

Three criteria were suggested for diagnosing IgG4-related CVD [8]: (1) Histologic findings of aortitis or periaortitis cannot be explained by another process, such as atherosclerosis. (2) > 50% of immunostained plasma cells are positive for IgG4. (3) The presence of > 50 IgG4 plasma cells per high-power microscopic field.

The main manifestation of cardiovascular involvement originates from the vessels, which are usually large, including the aorta and its major branches. In contrast, medium vessel involvement has been described less often. Vascular manifestations, including aortitis, arteritis, periaortitis, periarteritis, and inflammatory aneurysm, are well represented in the literature [2]. The less frequently reported cardiovascular manifestations include coronary artery involvement, pericardial involvement, myocardial involvement, and valvular heart disease [9].

Our case was representative of both pericardial and myocardial involvement. The pattern of myocardial injury was a nonischemic pattern with diffuse subepicardial late gadolinium enhancement, which also showed evidence of inflammation showing myocardial infiltrative involvement; the subendocardial layer remained intact, showing no evidence of infarction. This myocardial involvement coincided with visceral and parietal pericardial layer involvement, showing active inflammation in CMR. The cardiac involvement leads to constrictive physiology as septal bouncing and respiratory phasic interventricular interdependency. Also there was periarterial soft tissue infiltration around the coronary arteries.

The condition we present here, which involves perimyocarditis with evidence of constrictive pericarditis, is a rare manifestation of this multifaceted disease.

Previous studies have not reported the presence of calcification in IgG4-related disease. However, in our case, evidence of diffuse calcification was observed in the involved areas, including the pericardium, around the coronary arteries, and the mediastinum.

Herein, we review these rare IgG4-related cardiovascular manifestations. (Table 1).

**Table 1:** IgG4-related cardiovascular manifestations

Cardiovascular involvement	Imaging findings
1)Coronary artery involvement	Coronary stenosis, periarterial soft tissue infiltration, wall thickening, periarterial tumor-like lesions, coronary ectasia, aneurysmal formation
2)Pericardial involvement	Increased pericardial thickness, pericardial effusion, constrictive pericarditis
3)Myocardial involvement	Myocarditis, solitary or infiltrative myocardial masses
4) Cardiac cavity involvement	Intra-cavity mass-like lesions
5) Valvular heart involvement	Valvular stenosis or regurgitation, tumorous lesion on a valve leaflet

1.Coronary artery involvement.

IgG4-related coronary arteritis and periarteritis are uncommon but significant manifestations of IgG4-RD that can lead to severe complications such as myocardial infarction, ischemic cardiomyopathy, and aneurysmal rupture. This condition is typically observed in middle-aged to older male patients [10]. A systematic review conducted in 2022 analyzed 42 patients with IgG4-related coronary artery involvement [11]. The review revealed various manifestations of coronary artery involvement, including aneurysms or ectasia, stenosis, periarterial soft tissue proliferation, and arterial wall thickening. Mass-like or diffuse wall thickening was the most common presentation, with early-stage lesions possibly progressing to aneurysms or stenotic lesions. Many patients exhibit an extensive disease with multiple branch lesions [10, 11]. However, the location of coronary artery involvement does not show a significant pattern, and simultaneous involvement of other organs, particularly peri-aortitis, is commonly observed in these patients.

ECG-gated coronary computed tomography angiography (CTA) is an excellent diagnostic modality for IgG4-related coronary artery involvement, as it can provide a comprehensive view of the disease spectrum, including stenosis, periarterial soft tissue infiltration, wall thickening, tumor-like lesions, coronary ectasia, and aneurysmal formation [12]. A characteristic radiographic finding of IgG4-related coronary periarteritis in CTA is the presence of peri-arterial wall thickening and circumferential soft-tissue density. However, CTA may have limitations in evaluating the presence of stenosis in individuals with extensive coronary calcification [13]. Another non-invasive option for diagnosing coronary artery involvement is coronary magnetic resonance angiography, although its use may be limited due to a long imaging duration [14]. With the help of LGE tissue characterization, CMR can be valuable in detecting ischemic myocardial injury patterns secondary to coronary artery involvement in IgG4-RD [15].

Invasive (conventional) coronary angiography remains the gold standard method for assessing the severity of coronary stenosis and aneurysms.

Given the potential for serious cardiac complications in IgG4-related coronary arteritis and periarteritis, early and accurate diagnosis through appropriate imaging modalities is crucial. Awareness of this rare manifestation and its radiographic characteristics aids in guiding the optimal management and treatment decisions for affected patients.

2.Pericardial involvement.

IgG4-related pericardial involvement is a rare manifestation that has been reported sporadically. Identifying pericardial involvement in IgG4-RD is crucial for proper management and to avoid unnecessary interventions, such as cardiac surgeries or invasive procedures. A review conducted by Michaël Doumen et al. in 2021 included 32 patients with IgG4-related pericardial involvement [16]. Similar to other types of IgG4-RD, this condition is more commonly observed in older men. IgG4-related pericarditis was most frequently associated with pleural involvement, and it was mainly characterized by increased pericardial thickness and pericardial effusion. In some cases, it presented as constrictive pericarditis [17].

Diagnosing IgG4-related pericardial involvement often requires multimodality imaging. Echocardiography is typically the initial imaging test used. Contrast-enhanced CT images may reveal diffuse enhancing pericardial thickening and pericardial effusion as characteristic features of IgG4-related pericardial involvement. Combining the metabolic information from FDG PET/CT can be a powerful method for evaluating active inflammation [18]. Cardiac magnetic resonance (CMR) is the most comprehensive imaging modality, allowing for a detailed assessment of the heart's morphological features, pericardium, and hemodynamic characteristics. CMR can assess the extent of pericardial inflammation and provide valuable insights into constrictive pericarditis [19].

Due to the rarity of IgG4-related pericardial involvement and its potential clinical significance, physicians should remain vigilant and consider this condition in the differential diagnosis when evaluating patients with pericardial thickening, effusion, or constrictive pericarditis. Multimodality imaging techniques, such as echocardiography, CT, PET/CT, and CMR, can aid in accurate diagnosis and appropriate management of patients with IgG4-related pericardial involvement.

3. Myocardial involvement

Myocardial involvement in IgG4-related disease (IgG4-RD) is rare compared to the more commonly observed coronary artery and pericardial involvement. Myocardial involvement can manifest as myocarditis or the presence of solitary or infiltrative myocardial masses.

There have been reports of suspected IgG4-RD myocarditis based on findings such as FDG avidity on cardiac PET scans, evidence of inflammation on CMR, or steroid-responsive left ventricular dysfunction in patients with extracardiac IgG4-RD [20].

4. Cardiac cavity involvement as mass like lesion

In a study conducted in 2020 by Hajsadeghi et al., nine cases of pathology-confirmed IgG4-RD with intra-cardiac masses were reviewed. The right atrium was the most commonly affected cardiac structure by the mass [21]. The first step in diagnosing cardiac masses is typically echocardiography. However, CMR (Cardiac Magnetic Resonance) can provide a more precise diagnosis of cardiac masses in further workup. Employing T1-weighted, T2-weighted, and gadolinium-enhanced sequences by CMR enables thorough noninvasive tissue characterization [22]. In the context of IgG4-RD, myocardial lesions appear isointense in T1-weighted sequences and exhibit high signal intensity in T2-weighted sequences. Furthermore, these lesions demonstrate intense enhancement after the administration of gadolinium. In cases where CMR is unavailable, cardiac CT (Computed Tomography) can be used to assess heart masses. Cardiac CT offers advantages such as fast examination, high spatiotemporal resolution, and a wide field of view, which enables comprehensive evaluation of heart masses and the surrounding structures [23].

5. Valvular heart involvement

Valvular disease associated with IgG4-RD is exceptionally uncommon. The infiltration of IgG4-positive plasma cells can disrupt heart valve function, leading to stenosis and regurgitation. In some cases, IgG4 infiltration may result in the formation of a swollen tumorous lesion on a valve leaflet [24]. The specific implications of a diagnosis of IgG4-RD-associated valvulitis are not yet fully clear, and further research is needed to understand the clinical significance and management approach for this rare manifestation.

In cases where valve replacement is required, immunohistological analyses have shown the presence of IgG4-positive plasma cells in the excised valves. This highlights the importance of routine histological evaluation of surgically removed valves [25].

The treatment of IgG4-RD poses unique challenges due to its complex and rare nature. Current strategies aim to control the autoimmune response, reduce inflammation, and achieve sustained remission while minimizing glucocorticoid use. Glucocorticoids remain the first-line treatment for inducing rapid remission, but their long-term use requires careful monitoring due to potential adverse effects. For patients with inadequate response or relapses, immunosuppressive agents such as methotrexate, azathioprine, mycophenolate mofetil, and rituximab have shown promise as steroid-sparing alternatives. The relapsing nature of IgG4-RD necessitates long-term monitoring and adaptive treatment strategies to effectively manage the condition [26].

The presented case illustrates the complexity and challenges in diagnosing and managing IgG4-related disease. The initial clinical manifestations were non-specific, leading to a diagnostic journey involving various investigations and a breakthrough with the biopsy of a retroperitoneal mass showing increased IgG4-positive plasma cells. The patient responded well to the initial treatment with rituximab and prednisolone, but a recurrence of symptoms necessitated further investigation, leading to the diagnosis of diffuse pericardial and myocardial involvement.

The diagnostic criteria for IgG4-RD involve a combination of clinical, serological, radiological, and pathological features. Multimodality imaging, including echocardiography, CT, PET, and CMR, is crucial in identifying cardiac involvement and assessing disease severity.

Overall, greater awareness among healthcare professionals and further research into the various patterns of cardiac involvement in IgG4-related diseases are needed to improve diagnosis, management, and patient outcomes. Timely recognition and treatment can prevent potential organ damage and improve the overall prognosis of patients with IgG4-related diseases affecting the cardiovascular system.

## Data Availability

The data that support the findings of this study are available on request from the corresponding author. Ns.moosavi@yahoo.com.

## Abbreviations

IgG4-RD: Immunoglobulin G4-related disease
CT scan: Computed tomography scan
PET scan: Positron emission tomography scan
LV: Left ventricular
CMR: Cardiac Magnetic Resonance imaging
RV: Right ventricular
LVEDVI: Left ventricular end-diastolic volume index
RVEDVI: Right ventricular end-diastolic volume index
EF: Ejection fraction
LGE: Late gadolinium enhancement
CVD: Cardiovascular disease
CTA: Computed tomography angiography
